# Iron therapy in iron-deficiency patients with heart failure with preserved ejection fraction: A protocol for meta-analysis

**DOI:** 10.1097/MD.0000000000026919

**Published:** 2021-08-13

**Authors:** Hidekatsu Fukuta, Hiromi Hagiwara, Takeshi Kamiya

**Affiliations:** a Core Laboratory, Nagoya City University Graduate School of Medical Sciences, Nagoya, Japan; b Department of Medical Innovation, Nagoya City University Graduate School of Medical Sciences, Nagoya, Japan.

**Keywords:** anemia, heart failure, iron, meta-analysis

## Abstract

**Background:**

Nearly half of patients with heart failure (HF) have preserved ejection fraction (EF) and the mortality and morbidity of patients with HF with preserved EF (HFpEF) are high. However, there is no established therapy to improve survival in these patients. HFpEF patients are often elderly and their primary chronic symptom is severe exercise intolerance. Thus, improvement of exercise capacity presents another important clinical outcome in HFpEF patients. Iron deficiency is common in HF patients, and the presence of iron deficiency, regardless of concomitant anemia, is associated with worse symptoms, impaired exercise capacity, and higher mortality and hospitalization in these patients. Several meta-analyses of randomized controlled trials reported that iron administration improved HF symptoms, exercise capacity, and clinical outcomes in iron-deficiency patients with HF with reduced EF. However, there is insufficient evidence as to the effect of iron administration in iron-deficiency HFpEF patients.

**Methods and Results:**

This meta-analysis will include randomized controlled trials on the effects of iron administration on HF symptoms, exercise capacity, and health-related quality of life in iron-deficiency HFpEF patients. Information of studies will be collected from PubMed, Web of Science, Cochrane Library, and ClinicalTrials.gov. The primary outcome will be exercise capacity (6-minute walking distance). The secondary outcomes will be HF symptoms, health-related quality of life, and mortality and hospitalization rates.

**Conclusion:**

This meta-analysis will evaluate the effect of iron therapy in iron-deficiency HFpEF patients, providing evidence as to the iron administration in these patients.

**Systematic review registration:**

PROSPERO CRD42020205297.

## 1 Introduction

Nearly half of patients with heart failure (HF) in the community have preserved ejection fraction (EF) and the mortality and morbidity of patients with HF with preserved EF (HFpEF) are high.^[1–4]^ However, there is no established therapy to improve survival in these patients.^[5–9]^ Patients with HFpEF are often elderly and their primary chronic symptom is severe exercise intolerance.^[10,11]^ Thus, improvement of exercise capacity presents another important clinical outcome in HFpEF patients.

Iron deficiency is common in patients with HF with reduced EF (HFrEF), and the presence of iron deficiency, regardless of concomitant anemia, is associated with worse symptoms, impaired exercise capacity, and higher mortality and hospitalization in these patients.^[12–15]^ Multiple randomized controlled trials (RCTs) have examined the effect of iron therapy in iron-deficiency HFrEF patients.^[16–18]^ Several meta-analyses reported that iron administration improved HF symptoms, exercise capacity, and clinical outcomes in iron-deficiency HFrEF patients.^[19,20]^

It is accumulating evidence that iron deficiency is also common in HFpEF patients and that the presence of iron deficiency is associated with worse symptoms and impaired functional capacity in these patients.^[21]^ However, there is insufficient evidence as to the effect of iron administration in iron-deficiency HFpEF patients.

Accordingly, the purpose of this meta-analysis is to evaluate the efficacy as well as safety of iron administration in iron-deficiency HFpEF patients compared with standard therapy or control group.

## 2 Methods

This study has been registered as PROSPERO CRD42020205297 (https://www.crd.york.ac.uk/prospero/). This protocol for meta-analysis will be performed according to the Preferred Reporting Items for Systematic Review and Meta-analysis Protocols (PRISMA-P) statement.^[22]^

### 2.1 Search strategy

The electronic databases for literature search will include PubMed, Web of Science, Cochrane Library, and ClinicalTrials.gov. For search of the eligible studies, the following keywords and Medical Subject Heading will be used: *diastolic heart failure*, *heart failure with normal (preserved) ejection fraction, iron, anemia.* Only articles published in the English language will be included.

### 2.2 Study design

Only RCTs will be included. Observational cohort and case-control studies will be excluded.

### 2.3 Selection criteria

Inclusion criteria for this meta-analysis included: included patients with HFpEF; RCTs; administration of iron; compared with usual therapy or placebo control group; and assessed HF symptoms, exercise capacity, quality of life, morbidity, or mortality.

### 2.4 Outcomes

The primary outcome will be exercise capacity (6-minute walking distance). The secondary outcomes will be HF symptoms, health-related quality of life, and mortality and hospitalization rates.

### 2.5 Data extraction

Information on the study and patient characteristics, methodological quality, intervention strategies, and clinical outcomes will be systematically extracted separately by 2 reviewers. Disagreements will be resolved by consensus.

### 2.6 Quality assessment

The Cochrane Risk of Bias tool will be used to assess quality of RCTs included.^[23]^ The quality of evidence for the outcomes will be evaluated by the use of the Grading of Recommendations Assessment, Development and Evaluation (GRADE) system.^[24]^ The quality of evidence will be evaluated across the domains of risk of bias, consistency, directness, precision, and publication bias.

### 2.7 Statistical analysis

For continuous outcomes, the effect size for the intervention will be calculated by the difference between the means of the intervention and control groups at the end of the intervention. For morbidity and mortality, relative risk with 95% confidence interval will be calculated. For each outcome, heterogeneity will be assessed using the Cochran's Q and *I*^2^ statistic; for the Cochran's Q and *I*^2^ statistic, a *P* value of < .1 and *I*^2^ > 50% will be considered significant, respectively. When there is significant heterogeneity, the data will be pooled using a random-effects model; otherwise, a fixed-effects model will be used. Publication bias will be assessed graphically using a funnel plot and mathematically using Egger test. For these analyses, Comprehensive Meta Analysis Software version 2 (Biostat, Englewood, NJ) and STATA 16 software (Stata Corp LP, TX) will be used.

### 2.8 Sensitivity analysis

Meta-regression will be used to determine whether the effect of iron administration will be confounded by baseline clinical characteristics. Subgroup analysis stratified by route of iron administration (oral or intravenous) will be performed.

### 2.9 Ethical issues

This meta-analysis is a literature study. Ethical approval is not required because this meta-analysis will not involve any subject directly.

## 3 Discussion

Although recent meta-analyses on the effect of iron administration in HFrEF patients have reported the potential benefits,^[19,20]^ there is insufficient evidence as to the effect of iron therapy in HFpEF patients. To the best of our knowledge, this is the first meta-analysis protocol about iron therapy in iron-deficiency patients with HFpEF. The results will evaluate whether iron administration is beneficial for iron-deficiency patients with HFpEF, providing evidence regarding the iron administration in these patients.

## Author contributions

All authors critically revised the manuscript.

**Conceptualization:** Hidekatsu Fukuta.

**Data curation:** Hiromi Hagiwara, Takeshi Kamiya.

**Drafted manuscript:** Hidekatsu Fukuta.

**Funding acquisition:** Hidekatsu Fukuta.

**Literature retrieval:** Hiromi Hagiwara, Takeshi Kamiya.

**Methodology:** Hidekatsu Fukuta, Hiromi Hagiwara, Takeshi Kamiya.

**Supervision:** Takeshi Kamiya.

**Writing – original draft:** Hidekatsu Fukuta.

**Writing – review & editing:** Hidekatsu Fukuta, Hiromi Hagiwara, Takeshi Kamiya.
